# Neurophysiological differences between dexmedetomidine sedation and natural sleep across the rodent lifespan: implications for aging and perioperative brain health

**DOI:** 10.1007/s44254-025-00161-y

**Published:** 2026-01-19

**Authors:** Morgan J. Siegmann, Daniel P. Zachs, Jonathan D. Kenny, Arianna R. S. Lark, Fayaz A. Mir, Eric D. Melonakos, Mohsen Hozan, Sarah Toledano, Rebecca R. Goldblum, Yang Liu, Michael A. Nolan, Gabriella Cohen, Jinyoung Choi, Christian G. White, Eliza A. Crowley, Abigail Hardy Carpenter, Bryton A. Toro, Channing E. Syme, Emery N. Brown, Christa J. Nehs

**Affiliations:** 1 Mass General Brigham Department of Anesthesiology, Massachusetts General Hospital, Harvard Medical School, Boston, MA USA; 2 Division of Sleep Medicine, Harvard Medical School, Boston, MA USA; 3 Department of Brain and Cognitive Sciences, Massachusetts Institute of Technology, Cambridge, MA USA; 4 Picower Institute for Learning and Memory, Massachusetts Institute of Technology, Cambridge, MA USA; 5 Harvard–MIT Division of Health Sciences and Technology, Cambridge, MA USA; 6 Institute for Medical Engineering and Science, Massachusetts Institute of Technology, Cambridge, MA USA

**Keywords:** Spike wave discharge, Epileptiform activity, High voltage spindle

## Abstract

**Purpose:**

Dexmedetomidine is commonly used for its sedative and neuroprotective effects, but its impact on brain activity and sleep architecture is not fully understood. Emerging evidence suggests it may improve postoperative outcomes, particularly in older adults, by promoting sleep-like states with stable hemodynamics, reducing posttraumatic stress, and decreasing delirium. This study aims to better characterize the neurophysiological profile of dexmedetomidine-induced sedation by comparing it to natural sleep in both young and aged mice.

**Methods:**

Twelve 4–5 month old and six 10–18-month-old C57BL/6 J male mice were used. Animals were implanted with electroencephalography/electromyography electrodes. After at least 7 days of recovery, animals received intraperitoneal injections of saline or dexmedetomidine (50–400 µg/kg) and sleep–wake states were recorded for 5–12 h.

**Results:**

Dexmedetomidine significantly increased delta (0.5–4 Hz) power beyond levels observed during natural non-rapid eye movement (NREM) sleep, followed by suppression of both high frequency (> 10 Hz) electroencephalography activity and REM sleep in a dose dependent manner. Body posture was sprawled during dexmedetomidine versus curled as during natural sleep. Notably, at the transition into sedation, dexmedetomidine induced high-voltage spikes resembling high-voltage spindles and spike wave discharges. These spikes were more prominent in the prefrontal cortex compared to the parietal cortex and aged animals exhibited more high voltage spikes than young adult animals.

**Conclusion:**

The combination of elevated delta power, high-voltage spikes, suppression of high-frequency activity, and sprawled body posture during dexmedetomidine-induced sedation indicates a state of unconsciousness that is neurophysiologically distinct from natural NREM sleep in mice. These findings highlight important age-related differential responses to dexmedetomidine and help inform its safe and effective use in vulnerable patient populations.

## Introduction

Sleep is a naturally occurring, physiologically regulated behavioral state characterized by reduced responsiveness to external stimuli and distinct electrophysiological characteristics. It is essential for maintaining cognitive function, metabolic homeostasis, immune defense, and overall survival. In mammals, sleep alternates cyclically between non-rapid eye movement (NREM) and rapid eye movement (REM) stages [1]. These sleep cycles are crucial for the full spectrum of restorative processes that sleep provides. Chronic sleep deprivation or disruption has been associated with impaired cardiovascular [2], immune [3], and metabolic function [4], as well as cognitive deficits and increased disease risk.

Despite the prevalence of sleep disorders, current pharmacologic treatments often fail to reproduce the restorative features of natural sleep. Common sedative-hypnotics, such as benzodiazepine-like compounds, predominantly promote light NREM (stage 2) sleep and suppress REM sleep [5]. Newer agents, including dual orexin receptor antagonists, elevate NREM and/or REM sleep to varying degrees but still fall short of replicating the full architecture and functional benefits of natural sleep [6–8]. As a result, drug-induced sleep does not confer the same physiological recovery as spontaneous sleep [9–11].

Dexmedetomidine, a selective α2A adrenergic receptor agonist, is increasingly used in clinical settings as a sedative, particularly in the intensive care unit and perioperative care. It produces electroencephalogram (EEG) patterns resembling NREM sleep, including enhanced slow-wave (delta) power (0.5–4 Hz) and reduced muscle tone, observed across multiple species, including humans [12, 13], cats [14], rats [15], and mice [16]. Notably, dexmedetomidine also induces spindle-like activity that superficially resembles sleep spindles, though these events tend to be longer in duration [17, 18].

Anesthetics are thought to induce loss of consciousness, in part, through modulation of endogenous sleep–wake regulatory circuits [19, 20]. Dexmedetomidine is also thought to act at NREM sleep circuitry [5, 21, 22]. Specifically, dexmedetomidine binds to presynaptic α2A adrenergic autoreceptors in the locus coeruleus, decreasing norepinephrine release which disinhibits sleep-promoting regions such as the preoptic area [23]. Lesions in the preoptic area reduce the sedative effect of dexmedetomidine [21], and knockdown of α2A receptors in the locus coeruleus prevents the loss of the righting reflex at high doses while leaving low-dose effects (e.g., delta enhancement) intact [24]. These findings suggest that dexmedetomidine sedation recruits distinct neural circuits in a dose-dependent manner.

Despite many similarities to natural NREM sleep, accumulating evidence suggests that dexmedetomidine sedation is neurophysiologically and behaviorally distinct. For example, rats exhibit rebound increases in both NREM and REM sleep up to 48 h after a single dexmedetomidine injection, suggesting that the induced state does not fully satisfy homeostatic sleep needs [25]. Additionally, REM sleep is suppressed during dexmedetomidine sedation, and neural activation patterns, such as c-Fos expression in sleep-active regions of the basal forebrain and preoptic area, do not resemble those observed during natural sleep [26]. Behavioral differences are also evident: animals sedated with dexmedetomidine typically adopt a sprawled posture with open eyes, in contrast to the curled, eyes-closed posture seen during natural sleep [25].

These observations raise important questions about the nature of consciousness and restoration during dexmedetomidine sedation, particularly in older adults who are more likely to receive this agent perioperatively and are disproportionately vulnerable to postoperative cognitive dysfunction. In this study, we characterize the electrophysiological features of dexmedetomidine-induced sedation in both young and aged mice. We compare EEG and electromyogram (EMG) features across sedation and natural sleep states, focusing on time- and frequency-domain markers, including the emergence of high-voltage, spindle-like events during the transition to sedation. Our findings provide new insights into the neurophysiological distinctiveness of dexmedetomidine sedation compared to natural sleep and its age-dependent effects on brain activity.

## Materials and methods

### Animal care and use

We present this article in accordance with the ARRIVE reporting checklist [27]. Eighteen male C57BL/6JB6 mice from Jackson Laboratories were used, including 12 mice 4–5 months old and 6 mice 10–18 months old. A formal power analysis was performed using the MATLAB “samplesizepwr” function prior to study initiation. Based on published literature and preliminary pilot data, the resulting estimates informed the sample size used in this study. In alignment with the 3Rs principle (Replacement, Reduction, and Refinement), and it’s emphasis on reducing animal use, we selected the smallest sample size necessary to achieve adequate statistical power while minimizing the number of animals used. The smaller number of aged animals also reflects both limited availability and higher attrition rates during extended recording sessions, which are common challenges in aged animal research. Mice were housed individually with free access to food and water and kept on a 12:12 h light dark cycle (lights on at 7 a.m., lights off at 7 p.m.). All experiments were conducted during the day. All animal procedures adhered to ARRIVE (Animal Research: Reporting of In Vivo Experiments) Guidelines 2.0 and were approved by the MIT Committee on Animal Care (0511-044-14) or the MGH Subcommittee on Research Animal Care (2012N000030), with efforts made to minimize animal discomfort. All the aged mice in this study were sourced from the same inbred colony, housed under identical housing conditions, and underwent preliminary health assessments, including regular weight assessments, grooming behavior, and mobility checks to screen for overt signs of illness or advanced frailty before starting the experiments. Animals with evidence of major health impairments were excluded from the study. Although comprehensive standardized cognitive testing was not performed prior to inclusion, aged mice were visually screened for alertness and consistent baseline activity during pre-experimental acclimatization and baseline EEG recordings. Future studies could incorporate brief screening tasks to assess baseline cognition such as Y-maze spontaneous alternation (working memory), novel object recognition (recognition memory), and the open-field test (locomotion, exploratory drive, and anxiety-like behavior).

### Surgical implantation of EEG and EMG electrodes

Anesthesia was induced with 3% isoflurane in oxygen and maintained at 1.5% isoflurane in oxygen. A heating pad maintained the animal’s body temperature at 37° C throughout the surgery. Mice were surgically implanted with a preassembled implant consisting of an 8-channel Neuralynx electrode interface board with 6 EEG electrodes (0.005″ stainless steel, A-M Systems, Sequim, WA) and 2 EMG electrodes (0.002″ 7-stranded stainless steel, A-M Systems, Sequim, WA). A microdrill was used to make 3 craniotomies for EEG electrodes over the prefrontal cortex (2.5 mm anterior; 1 mm lateral to Bregma), parietal cortex (2.5 mm posterior; 2 mm lateral to Bregma) and cerebellum (−6 mm posterior, 2 mm lateral to Bregma) and 6 craniotomies for anchor screws and a ground screw (0.7 mm diameter, 2 mm long, Antrin Miniature Specialties, Inc., Fallbrook, CA). The 2 EMG electrodes were placed between the nuchal muscles. The electrodes and screws were affixed to the skull with dental acrylic. The electrodes were connected to an 8-channel Neuralynx electrode interface board. EEG signal quality was assessed prior to experimental recordings by acquiring baseline EEG to verify low noise levels, stable electrode contacts, and absence of excessive drift or artifacts. Only animals that met these signal quality criteria were included in the study. We ensured secure and stable connections between electrodes, the animal, and the acquisition board. Data were sampled at 500 Hz and filtered with a 0.5 Hz high-pass and 40 Hz low-pass filter to optimize signal quality. All animals postoperatively received the analgesic ketoprofen (4 mg/kg) every 24 h as needed until recovered fully. Animals were allowed a minimum recovery period of 7 days prior to experimentation.

### Dexmedetomidine administration and electrophysiology

Experiments started between 9 and 10 am. For each recording, a minimum of 10 min of EEG and EMG during baseline wakefulness was recorded. Following the baseline recording, an intraperitoneal (IP) injection of saline (vehicle), 50, 100, 200, or 400 µg/kg dexmedetomidine HCl (Precedex, Abbott Laboratories) was administered. Our chosen doses (50–400 µg/kg, IP) were selected based on previous literature [24, 25] with the goal of spanning a range from mild sedative effects to near-maximal effects seen at 200–400 µg/kg. At the lower doses (50–100 µg/kg), animals remained arousable, whereas at 200–400 µg/kg they were not readily arousable. These IP bolus doses are not directly comparable to the low-dose continuous intravenous infusions used clinically in humans, but they fall within the established sedative range for rodent studies and were selected to capture both the ascending and plateau phases of the dexmedetomidine dose–response curve. The injection volume was 5 ml/kg. A heating pad was placed underneath the animal during dexmedetomidine anesthesia to maintain body temperature. The mice behaved freely while EEG (filtered 0.5–500 Hz), EMG (filtered 10–500 Hz), and video were recorded for 5–12 h using a 64-channel Neuralynx Digital recording system at a sampling rate of 500 Hz (Neuralynx Inc., Bozeman, Montana). The EEG, EMG, and video were used to visually score wakefulness, NREM sleep, REM sleep, and dexmedetomidine-induced sedation in 2 s epochs in Spike2 (CED, Cambridge, UK). The animals were monitored until normal sleep and wakefulness had returned as indicated by the EEG and EMG. The animals were conditioned to the recording chamber prior to the experiment. There were at least 3 days of rest between consecutive experiments.

### High-voltage spike event detection

At the onset of dexmedetomidine-induced sedation, there were large amplitude spike waveforms in the EEG of both young and aged mice. Below is our method to quantify the timing of the spike events. Inter-animal variability in the magnitude of the recorded signal made a universal amplitude threshold for event detection impractical. The MATLAB function *findpeaks* was employed to find local maxima. Each recording had a threshold so that only peaks in the top 99th percentile of the raw signal could be considered candidate spike events. To better distinguish all high-amplitude EEG content from dexmedetomidine-induced spike waves, candidate spike event waveforms were required to have a width at half height between 20 and 120 ms. This range was determined by measuring the width of exemplary dexmedetomidine-induced spikes. To increase this method’s discriminatory power, the width at 0 mV was measured and required to fall between 40 and 260 ms. The dexmedetomidine spikes were observed to be narrow relative to height (measured peak to trough); therefore, a minimum height to width ratio of 3.25 µV/ms was imposed to further increase specificity. For the same reason, the ratio of width at half height and width at zero was measured, eliminating candidate events if the width at zero was more than 4.5 times greater than the width at half height (see Fig. 6A). This method for spike detection fundamentally relied on finding peaks before measuring features of them, as described above. Because some EEG recordings featured high-amplitude negative peaks, the method described above was performed twice, once for positive peaks and once for negative peaks. Positive and negative peaks within 120 ms of each other likely belong to the same waveform; therefore, one was considered a duplicate and was not counted. Group-level spike rate over time was estimated using a moving average of the event raster with a window size of 1 min. A two-tailed, two-sample t-test on the number of spikes during the first 10 min post-injection was used to compare spike activity between age groups within each dose condition. Multiple comparisons were corrected for using the Bonferroni correction resulting in alpha = 0.05/4 = 0.0125.

### EEG spectral comparison of dexmedetomidine-induced sedation to NREM sleep

The spectral content of the EEG response to a bolus dose of dexmedetomidine changes gradually over time. In order to capture the different phases of dexmedetomidine-induced sedation, 2epochs of 2-min duration were selected from each dexmedetomidine recording. The first 2 min of continuously sedated behavior, which started 5 min or 3 h after injection of dexmedetomidine, were selected for the early and late epochs, respectively. For each epoch the power spectrum was estimated from the time-averaged spectrogram over a range of 0 to 30 Hz, computed using the multitaper method (Chronux toolbox) with a 10 s, non-overlapping window, a time-bandwidth product of 6, and 11 discrete prolate spheroidal sequences (DPSS) tapers, normalized by the mean baseline power.

For pairwise comparison of dexmedetomidine and saline conditions, the power spectrum for each animal’s saline condition was estimated exclusively for NREM periods using the same spectral estimation parameters as above. The resulting power spectra were averaged over time and converted to decibels. The mean NREM power spectrum for each animal was normalized by its own mean baseline power and subtracted from each corresponding dexmedetomidine dose condition. This collection of spectral differences was sampled with replacement N times, where N is the number of animals that received the respective condition, and the median was taken. From this collection of median spectral differences, 95% confidence intervals (CIs) were bootstrapped using 10,000 samples.

### Delta power comparison to NREM sleep

To measure the changes in delta power over time, the first 4 h after dexmedetomidine administration were analyzed. The delta power was estimated using multitaper methods (Chronux toolbox) with a non-overlapping window length of 40 s, a time bandwidth product of 6, 11 DPSS tapers, and a frequency range of 0.5 to 4 Hz. The power spectra were integrated with respect to frequency and normalized by the mean baseline power. NREM sleep periods during the saline injections were processed in the same way and averaged over time. The dexmedetomidine condition delta power was divided by the mean saline NREM sleep delta power and converted to decibels. This collection of spectral differences was sampled with replacement N times for each animal in the condition and the median was taken. From this collection of median spectral differences, 95% CIs were bootstrapped using 10,000 samples.

### Statistical analysis

All statistical analyses were performed using custom scripts in MATLAB, Python, or Prism. Data were assessed for normality using descriptive statistics prior to inferential testing. The choice of statistical test was guided by data distribution and experimental design. To account for the unequal group sizes between young (n = 12) and aged (n = 6) animals, we employed nonparametric, bootstrap-based statistical approaches that are robust to differences in sample size and distributional assumptions. Group differences were considered statistically significant at *P* < 0.05 or by taking the difference of the 95% bootstrapped CIs where the difference did not cross zero. CIs were derived from 10,000 resamples, and effect sizes were calculated to assess the magnitude and reliability of group differences (see [28] for details). These methods provide stable estimates even with smaller sample sizes and help mitigate potential biases associated with unequal group numbers. Percent time sedated and percent time in REM sleep data were assessed via two-way analysis of variance (ANOVA, type III sum of squares; Assumptions: normality, homogeneity of variance) followed by post hoc Tukey’s multiple comparisons test. We assessed spectral differences between sedation and natural NREM sleep using a nonparametric bootstrapped 95% CI approach for the group median spectra. Frequency ranges were deemed significant when their CI did not include zero. To assess changes in delta power (0.5–4 Hz) following dexmedetomidine sedation, we calculated group medians and bootstrapped 95% CI at each time point. Differences between groups were deemed significant where confidence intervals did not overlap. Interspike intervals were compared between groups using the Wilcoxon rank-sum test due to non-normal distributions. Total spike counts were analyzed using two-tailed, two-sample t-test, also corrected for multiple comparisons with a Bonferroni-adjusted α = 0.0125.

## Results

### Dexmedetomidine-induced dose-dependent sedation and suppressed REM sleep

**Fig. 1:**
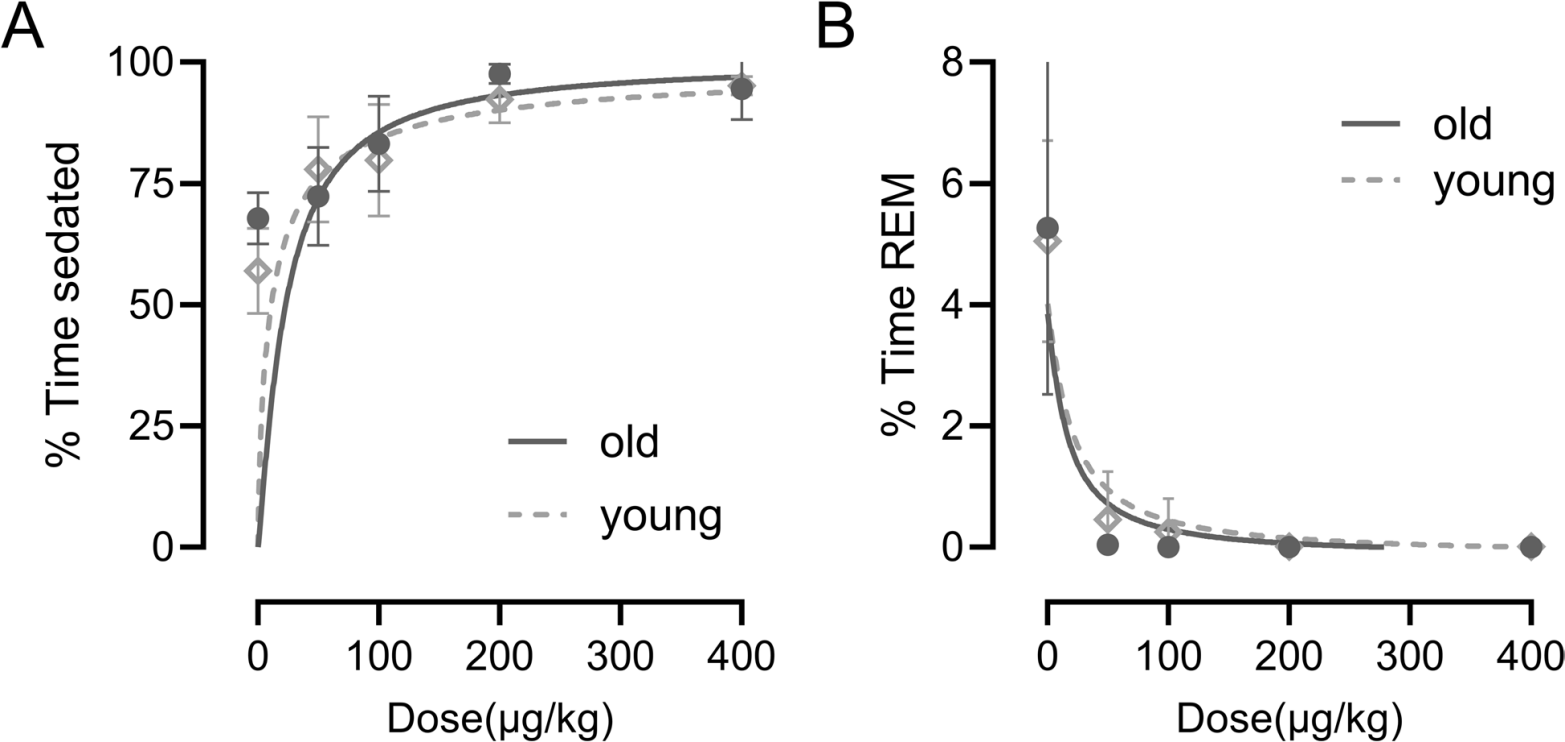
Dexmedetomidine produced sedation (**A**) and REM sleep suppression (**B**) in a dose dependent manner. Percent time sedated and REM sleep were calculated cumulatively for the first 4 h post-dexmedetomidine administration and averaged for young adult (**A**, n = 12) and aged mice (**B**, n = 6) by dose. While REM sleep suppression appeared to be more profound in aged mice due to the extremely minimal REM in both young and old there were no significant differences as determined by a two-way ANOVA. There was no significant difference in total sedation time between young adult and aged mice, as determined by a two-way ANOVA (type III sum of squares). Within each age group, all dose comparisons showed significant differences except between 0 vs. 50 µg/kg and 200 vs. 400 µg/kg, based on Tukey’s multiple comparisons test. Data are plotted using Prism’s dose response curve nonlinear least squares fit as mean ± standard deviation. *ANOVA* analysis of variance, *REM* rapid eye movement

Sedation was quantified by manually scoring the EEG for increased delta power and EMG for decreased muscle tone in 2-s epochs. Figure 1A illustrates the duration of sedation produced by different doses of dexmedetomidine in young adult and aged mice. In the first 4 h after dexmedetomidine administration, there was no significant difference in the amount of time spent sedated between young and aged mice (type III sum of squares two-way ANOVA). Post hoc Tukey multiple comparisons of the group means using combined age groups for dose revealed all dose combinations had significantly different means, except doses 0 vs 50 and 200 vs 400 µg/kg. Figure 1B shows dexmedetomidine suppression of REM sleep compared to the saline control. In aged mice, REM sleep was completely suppressed for all doses above 50 µg/kg for the first 4 h. The aged mice cumulatively experienced only three REM sleep episodes from all dexmedetomidine sedation conditions with no individual mouse experiencing more than one in the first 4 h. Young mice had a few more REM sleep episodes with low dose dexmedetomidine but still fewer than the saline controls.

### Dexmedetomidine increased delta power, followed by suppression of high frequency power

**Fig. 2:**
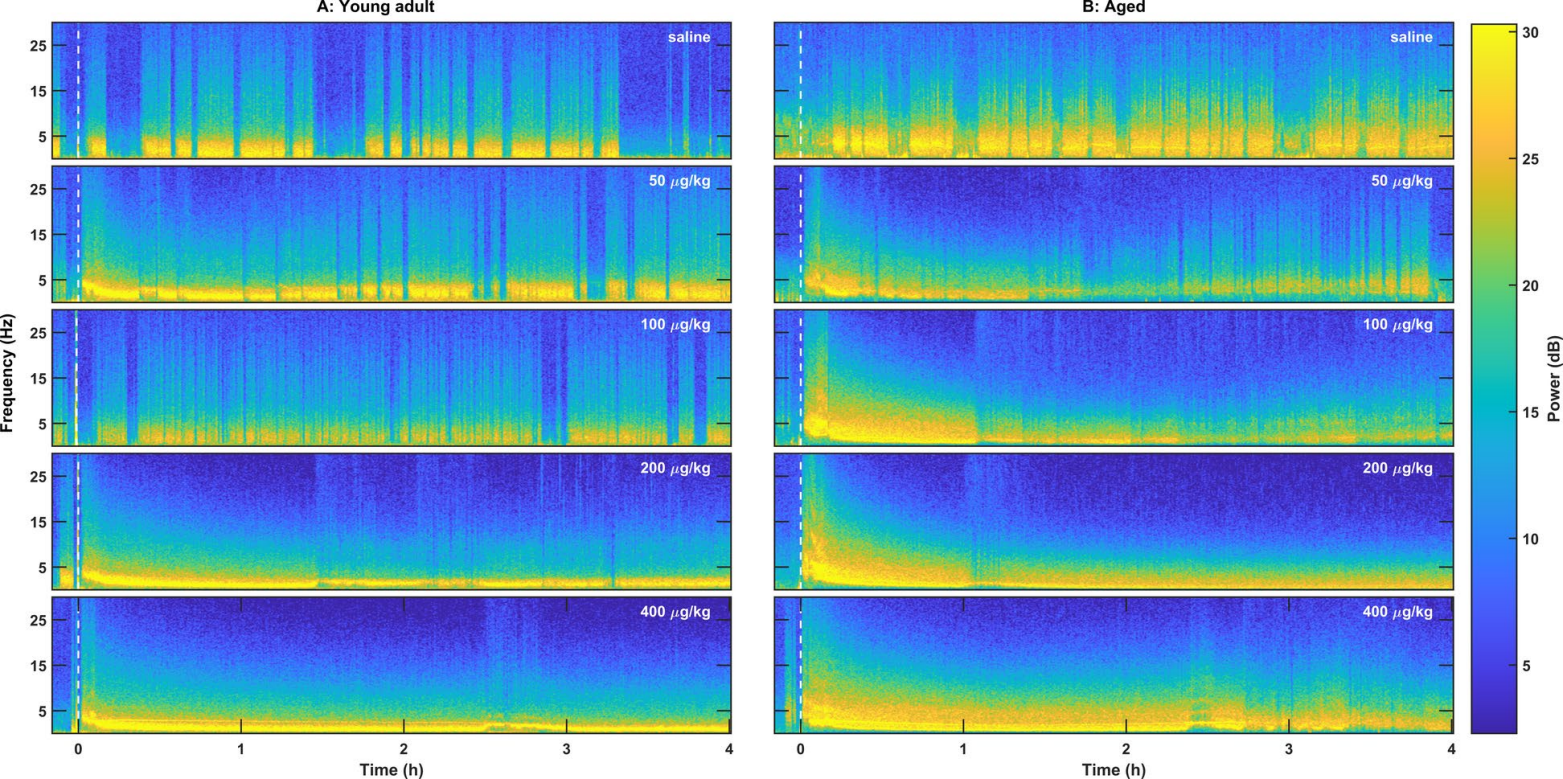
Bolus intraperitoneal injection of dexmedetomidine produced dose-dependent changes in the EEG oscillations in young adult and aged mice. **A** Representative spectrograms from a young adult mouse showed power concentrated in the low delta range following dexmedetomidine administration. **B** Representative spectrograms from an aged mouse showed high-frequency harmonics from high-voltage spikes immediately following dexmedetomidine administration and power spread over a large frequency range over the first hour. The dashed white line at time = 0 indicates dexmedetomidine or saline administration. Multitaper spectral estimation was performed using a window size of 40 s, a step size of 10 s, and 11 DPSS tapers. *DPSS* discrete prolate spheroidal sequences, *EEG* electroencephalogram

**Fig. 3:**
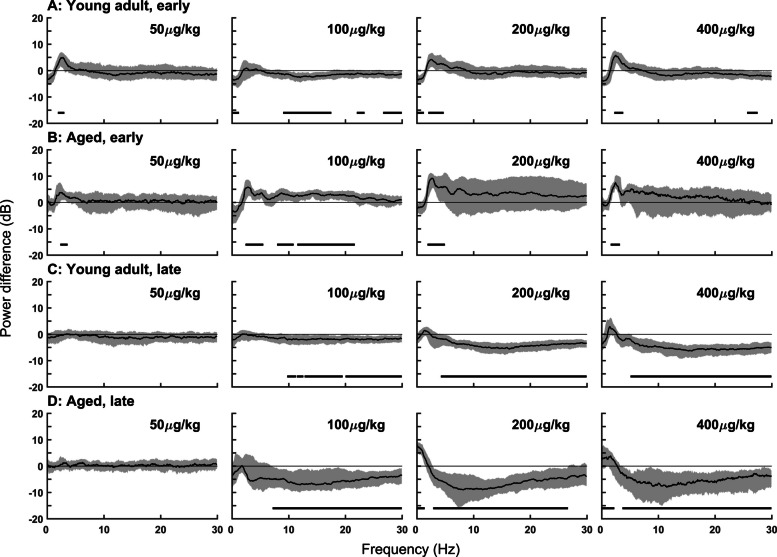
Dexmedetomidine increased delta power initially (early) and attenuated high-frequency power (later) compared to NREM sleep. Spectral differences between dexmedetomidine sedation and NREM sleep in young adult and aged mice were calculated using 2-min epochs taken from 5 min (**A** and **B**) and 3 h (**C** and **D**) post-dexmedetomidine injection. Power spectra were estimated using multitaper methods. To assess spectral differences between sedation and natural NREM sleep, we applied a nonparametric bootstrap approach to generate 95% CIs for the group median spectra. Frequency ranges where the CIs of the difference did not include zero were considered statistically significant. These significant regions are indicated by horizontal black bars at the bottom of each graph. CI confidence interval, *NREM* non-rapid eye movement

Dexmedetomidine produced an initial increase in delta power (0.5–4 Hz), followed by a gradual decrease in the frequency of delta power over the first hour. This change in frequency dynamics with time was best observed in the multitaper spectrogram (Fig. 2). The reduction in the frequency of the strong delta oscillation was accompanied by attenuation of frequencies above 10 Hz. The duration of increased delta power and high-frequency attenuation was dose dependent and extended for hours. In order to quantify the spectral qualities of dexmedetomidine-induced sedation, 2-min epochs located 5 min or 3 h post dexmedetomidine administration were selected for pairwise spectral comparison to natural NREM sleep. In both young and aged mice, early epochs after dexmedetomidine administration had consistently higher delta power than natural NREM sleep (Fig. 3). Exact frequency ranges of statistically significant increased power follow: 50 µg/kg: young 1.7–3.1 Hz, aged 1.7–5.1 Hz; 100 µg/kg: young ns, aged 2.1–6.0 Hz; 200 µg/kg: young 1.8–4.9 Hz, aged 1.7–5.4 Hz; 400 µg/kg: young 1.9–4.1 Hz, aged 1.4–5.5 Hz. Three hours after dexmedetomidine administration, there was significantly lower power in high frequencies compared to NREM sleep in all doses except 50 µg/kg in both young and old mice. Exact frequency ranges of significantly reduced power compared to NREM sleep follow: 100 µg/kg: young 8.9–30 Hz, aged 2.6–30 Hz; 200 µg/kg: young 4.1–30 Hz, aged 2.5–30 Hz; 400 µg/kg: young 4.8–30 Hz, aged 3.0–30 Hz. Additionally, in the aged mice 3 h after dexmedetomidine administration, 200 and 400 µg/kg dexmedetomidine produced increased slow/delta power in the range of 0–1.6 and 0–2.2 Hz, respectively.

### High doses of dexmedetomidine resulted in greater delta power compared to NREM sleep in young and aged mice

**Fig. 4:**
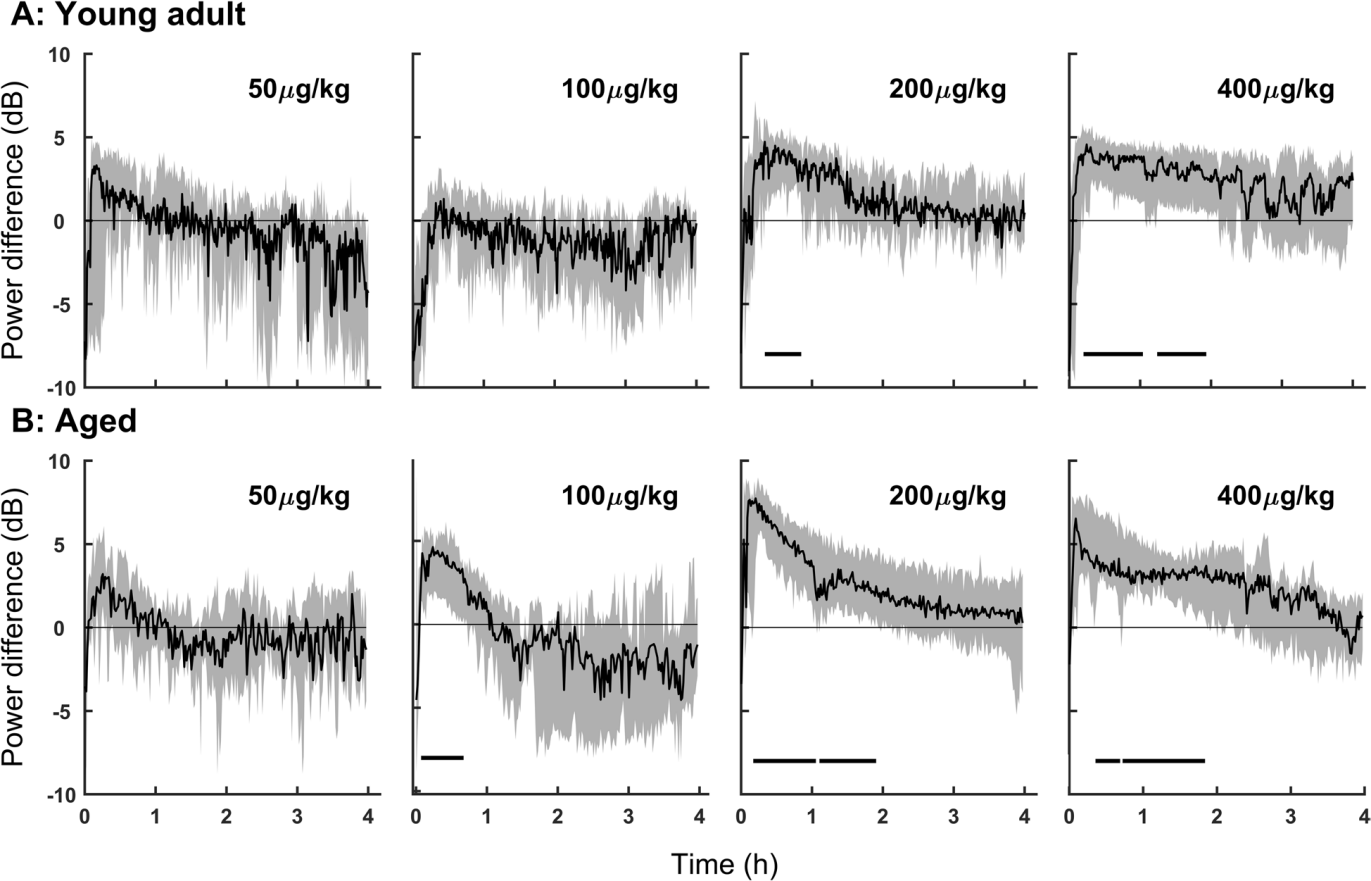
Dexmedetomidine induced a stronger and longer-lasting increase in delta power compared to natural NREM sleep in aged mice. Time course of spectral differences in delta power after dexmedetomidine compared to delta power during NREM (saline condition) over the first 4 h of the recording in young (**A**) and aged (**B**) mice. Delta power was estimated using multitaper methods. To assess spectral differences between sedation and natural NREM sleep, we applied a nonparametric bootstrap approach to generate 95% CIs for the group median spectra. Frequency ranges where the CIs of the difference did not include zero were considered statistically significant. These significant regions are indicated by horizontal black bars at the bottom of each graph

Dexmedetomidine administration increased delta power, as evidenced by the spectral analysis detailed above. To investigate the timecourse and magnitude of this increase, delta power during each animal’s dexmedetomidine condition was compared to the mean NREM sleep delta power in the saline condition for the first 4 h of the recording. Higher delta power was observed in the dexmedetomidine condition for almost all doses and was statistically significant (the difference of the bootstrapped 95% CIs did not overlap 0) in aged mice for doses 100, 200 and 400 µg/kg and young mice for doses 200 and 400 µg/kg (Fig. 4). The magnitude and consistency of this increase in delta power was dose dependent, such that higher doses more consistently resulted in a greater and longer difference from natural NREM delta power. It is well known that aging has significant effects on sleep–wake architecture in rodents, characterized by increased delayed latency to sleep, sleep fragmentation, decreased sleep consolidation, and alterations in EEG signatures, including weakened delta power especially during NREM sleep [29, 30]. Aging reduces neuronal responsiveness to sedative drugs, likely due to less sensitive alpha-2 receptors, altered ion channels, and more oxidative stress in older animals [31, 32].

### Characterization of high-voltage spikes induced by dexmedetomidine display age-specific differences

**Fig. 5:**
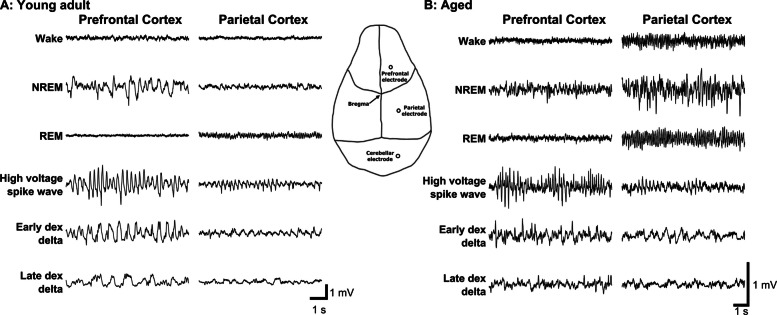
Dexmedetomidine produced electrophysiological signatures distinct from natural sleep and wake states in young adult and aged mice. The overall amplitude of the EEG in aged mice was smaller (zoomed in vertically to see the oscillations better) compared to young adult mice. Following dexmedetomidine administration, high-voltage spikes appear in the prefrontal cortex EEG and are lower in amplitude in the parietal cortex, see inset diagram for electrode locations. High-voltage spikes gradually reduce in frequency and form high-amplitude delta waves early in the recording, transitioning later into lower amplitude delta waves with the high frequencies attenuated. Panel **A** shows comparisons between prefrontal cortex and parietal cortex channels from the same young adult mouse under different sleep and dexmedetomidine induced states. Panel **B** shows the same channels in an aged mouse

**Fig. 6:**
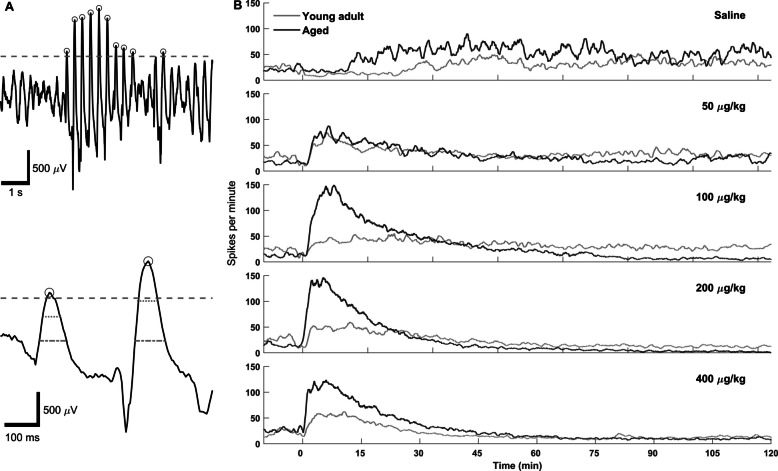
Dexmedetomidine induced high-voltage spikes, especially in aged mice. High-voltage spikes were detected based on waveform shape and amplitude of individual peaks. **A** (top panel) shows waxing and waning amplitude of typical high-voltage spikes from a young adult mouse. **A** (bottom panel) shows two magnified spikes from the above waveform. The grey dashed line indicates the 99th percentile amplitude threshold, the dotted line indicates peak width measured at half height, the dash-dotted line indicates peak width at zero microvolts, and the circles indicate peaks that have met the spike criteria. **B** Time course of spike activity following dexmedetomidine administration in a representative young and aged mouse. Traces show the number of spikes per minute over the first 2 h following a bolus injection of dexmedetomidine or saline (time = 0). Data illustrate age-related differences in the temporal dynamics of spike occurrence during the early post-injection period. Spikes per minute were calculated using a moving average with a window size of 1 min

**Fig. 7:**
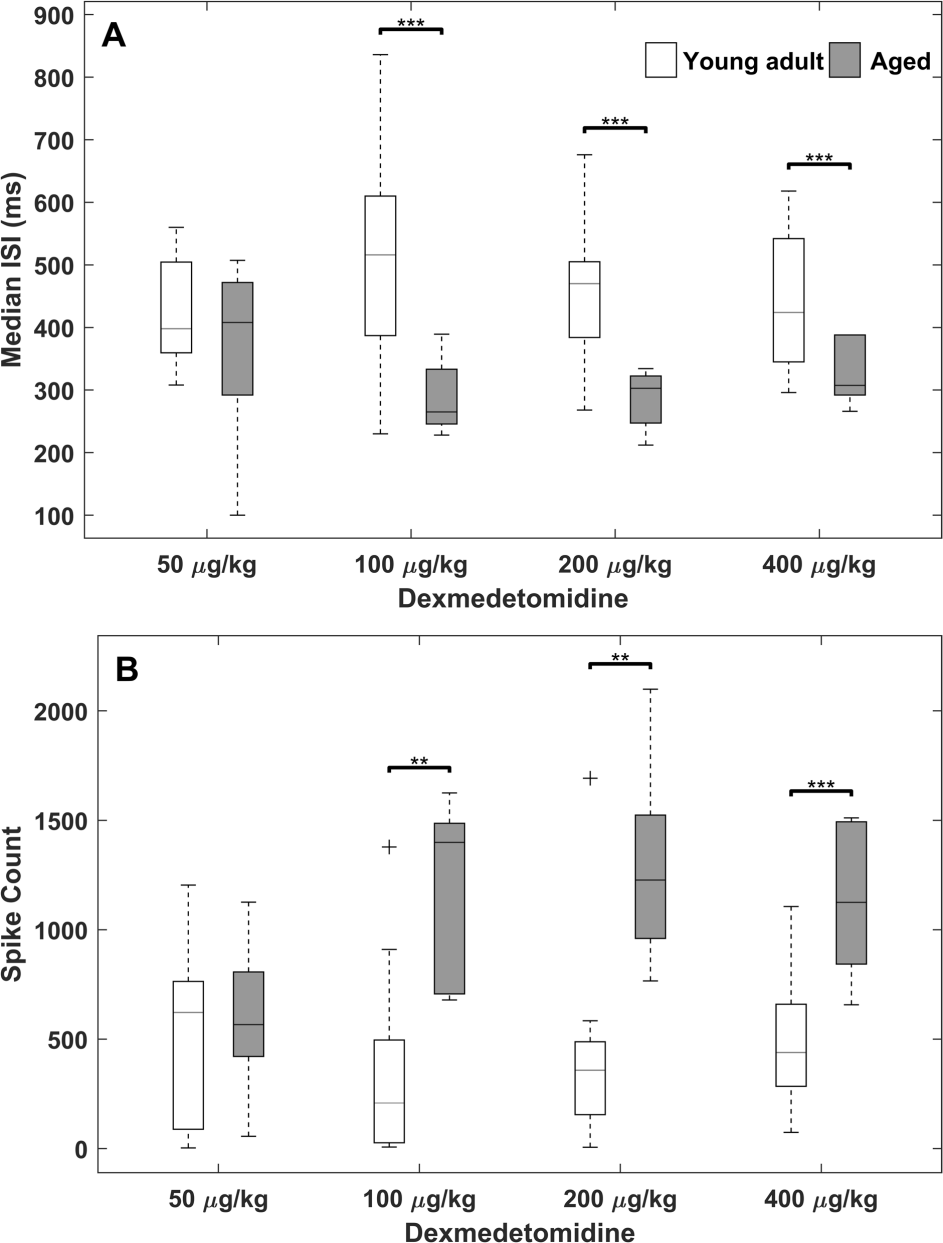
Young adult mice had higher interspike intervals and fewer spike events than aged mice for doses above 50 µg/kg. Group data taking the spikes from the first 10 min following dexmedetomidine administration were used for this comparison. **A** Wilcoxon rank-sum test was used to test for significance between young and aged interspike intervals (ISI) (alpha = 0.0125). **B** A two-tailed, two-sample *t*-test was used to test for significance between young and aged spike counts (alpha = 0.0125). Center lines indicate median, box edges indicate 1st and 3rd quartile, whiskers indicate range, and “ + ” indicates outliers. ** *P* < 0.01, *** *P* < 0.001

A bolus (IP) dose of dexmedetomidine produced bursts of high-voltage spiking activity in the EEG at the onset of sedation and immediately following subsequent arousals. Dexmedetomidine-induced spikes are high voltage, narrow, and come to a sharp peak. In both young and aged mice, dexmedetomidine spikes have the highest amplitude in the prefrontal cortex channel (Fig. 5) and have consistent onset patterns. Spikes typically start as early as a few minutes post-injection, often beginning before loss of muscle tone and continuing during the transition into the sedated state with low muscle tone. Initially, dexmedetomidine spikes come in bursts lasting less than a second, with subsequent bursts lasting longer and eventually merging into one long spike train. Alongside this shortening of the inter-burst interval, the interspike interval within bursts gradually increases. As the frequency of the spikes drops, this coincides with spikes becoming less narrow and transitioning into high-amplitude delta waves. While spikes were consistently observed at the beginning of sedation, clear spikes were observed as late as 3.5 h post dexmedetomidine administration. In these cases, spikes followed short arousals, indicated by an increase in muscle tone. While the basic characteristics of spikes were similar between young and aged mice, the spikes per minute appeared to vary in the two groups. This is evident in the spike rate over time, estimated using a simple moving average with a 1-min window (Fig. 6). To test this observation statistically, spikes were counted in the first 10 min of each recording and compared between young and aged animals in each condition. For doses 100, 200, and 400 µg/kg, aged mice had significantly more spikes than young mice (*P* < 0.005, 0.005, and 0.001, respectively). The interspike interval was compared between young and aged, using the same 10-min sample from the beginning of the recording (Fig. 7). Aggregate interspike intervals from each age-dose condition were compared using a Bonferroni-corrected rank-sum test due to the non-normal distribution of interspike intervals. Young mice had statistically significantly longer interspike intervals than their aged counterparts for doses 100 (young: 516, aged: 265 ms), 200 (young: 470, aged: 302 ms), and 400 µg/kg (young: 424, aged: 307 ms). Aged mice likewise had greater numbers of spikes than young mice for doses 100, 200, and 400 µg/kg. In addition, our lab also observed similar high-voltage spikes in young and old rats given 300 µg/kg dexmedetomidine via IP injection (unpublished observation), suggesting that these dexmedetomidine-induced spikes are not unique to mice.

Because aging is associated with well-documented changes in baseline EEG dynamics, including delta power and spike incidence, the between-group comparisons in Fig. 7 are intended to capture how dexmedetomidine interacts with these age-related physiological differences, rather than to normalize them away. These baseline differences are intrinsic to the biological processes under study and are critical for understanding age-dependent responses to sedation. Although the aged group had a smaller sample size, the observed age-related differences were consistently larger than within-group variability, and effect sizes were large across the key measures. These findings were further supported by convergent changes in multiple physiological features including spectral power, number of spikes, and inter-spike interval, underscoring the robustness of the effects.

## Discussion

### Dexmedetomidine induced dose dependent sedation and REM sleep suppression in addition to high voltage spike waves that increased with age

Our results align with previous studies, demonstrating that dexmedetomidine induces spectral changes in the EEG, as well as alterations to sleep architecture and related phenomena that do not fully resemble natural sleep. Specifically, we observed dose-dependent sedation and suppression of REM sleep (Fig. 1), consistent with prior work [22, 33]. Young mice exhibited extensive but incomplete REM suppression at lower dexmedetomidine doses, whereas aged mice showed near-total REM loss, suggesting that aging increases susceptibility to REM suppression beyond the drug’s direct effects. This may reflect reduced REM resilience or broader age-related changes in sleep regulatory circuits. However, high-dose dexmedetomidine led to EEG changes distinct from natural NREM sleep, such as high-voltage spikes, increased delta power, and an initial increase followed by suppression of high-frequency activity (Fig. 2). Notably, these changes in delta power (Figs. 3 and 4) and high-voltage spikes (Figs. 5 and 6) differed between young adult and aged mice, highlighting the influence of age on dexmedetomidine’s effects.

### Temporal dynamics of delta oscillations under dexmedetomidine

A progressive shift toward lower delta frequencies was observed following dexmedetomidine administration (Figs. 2, 3, and 4). Similar temporal dynamics have been described during natural sleep, where delta activity slows and decreases in amplitude as sleep progresses [34]. This shift likely reflects a combination of pharmacokinetic and physiological mechanisms. After bolus injection, high drug concentrations initially produce strong cortical synchronization, which evolves as concentrations decline over time. Human EEG studies have shown that as dexmedetomidine concentration increases, lower-frequency activity becomes progressively dominant while higher frequencies are suppressed [35–37], consistent with a gradual spectral slowing. Our data suggest that this suppression of higher frequency activity is dose-dependent- likely corresponding to the depth of sedation achieved. Delta in this case is comparable to baseline NREM sleep levels at 3 h. In Fig. 4, higher bolus doses of dexmedetomidine appear to drive delta enhancement (relative to baseline NREM) for modestly longer however these dynamics are likely different in continuous infusion. Beyond dexmedetomidine, similar dynamics have been documented under propofol anesthesia. In humans slow-wave frequency is continuously modulated over time during anesthetic state transitions, quantified using a “slow modulation index” [38]. Another study likewise showed that as anesthesia deepens, slow-oscillation cycle durations broaden, indicating a shift toward slower rhythms [39]. These findings provide a strong precedent for interpreting the downward drift in delta frequency under dexmedetomidine as a physiologically meaningful phenomenon. In addition to pharmacokinetics, intrinsic network mechanisms likely contribute. Cortico-thalamic oscillations are shaped by slow K⁺ currents, h-currents, and synaptic adaptation, which can modulate oscillatory pace over time [40]. Dexmedetomidine also recruits endogenous sleep-promoting circuits [21, 24], and the observed spectral evolution may reflect transitions within these circuits toward states dominated by slower oscillatory components, paralleling the natural progression of NREM sleep.

### Dexmedetomidine-induced high-voltage spikes resemble high-voltage spindles

Dexmedetomidine not only induced dose-dependent sedation (Fig. 1) but also led to the emergence of high-voltage spikes in the EEG (Fig. 5). Additionally, previous studies on adrenergic mechanisms of spike-wave discharges in epileptic WAG/Rij rats showed that dexmedetomidine can trigger both genuine spike-wave discharges and rudimentary epileptiform activity in susceptible rats. These spikes resemble the spike morphology and dynamics observed in our mice [41]. These findings, along with our unpublished observations of spikes in both young and aged wild-type rats following dexmedetomidine administration, suggest that the mechanism underlying dexmedetomidine-induced spikes is consistent across species.

Microinjection studies targeting the thalamus have shown that α2-adrenergic agonists such as xylazine and clonidine increase the occurrence of high-voltage spindles, similar to our observations with dexmedetomidine [42]. The high-voltage spikes observed in the present study closely resemble both natural and pharmacologically induced high-voltage spindles described by Buzsaki [5, 42], particularly in terms of waveform morphology, onset dynamics, age-related prevalence, and regional distribution. In freely behaving rats, high-voltage spindles have been detected during quiet wakefulness, with aged rats exhibiting increased incidence and longer durations [5]. These high-voltage spindles were highly synchronous across hemispheres and had the largest amplitude in posterofrontal-parietal cortical regions. Notably, their frequency typically began at 8–10 Hz and progressively slowed to 6–8 Hz. Our findings in mice are broadly consistent with this description, especially in terms of topography and temporal evolution, though the observed frequencies were lower. Specifically, we noted a similar gradual slowing of intraspike frequency, from approximately 5–6 Hz to 3–4 Hz over time. This species-specific difference in frequency may reflect variations in cortical circuit dynamics between mice and rats or differential sensitivity to dexmedetomidine across species.

### Potential mechanisms underlying dexmedetomidine high-voltage spikes

The high-voltage spikes observed in our study likely arise from two converging actions of norepinephrine modulation, primarily within the thalamus but expressed across the cortico-thalamo-cortical network. First, GABAergic neurons in the reticular thalamic nucleus, which act as pacemakers, rhythmically hyperpolarize thalamocortical relay neurons, generating synchronized bursting activity. Under normal conditions, noradrenergic input from the locus coeruleus suppresses this oscillatory behavior by promoting tonic firing in thalamocortical neurons. Dexmedetomidine, acting through presynaptic α2A autoreceptors, suppresses locus coeruleus activity and reduces norepinephrine release, thereby disinhibiting thalamic burst firing and promoting synchronized oscillations in the cortico-thalamo-cortical loop [23].

Building on this framework, Sitnikova et al. (2023) proposed that dexmedetomidine shifts the balance between α1 and α2 adrenergic receptor activity within the thalamus. Whereas α1 receptors generally promote cortical desynchrony and exert anti-epileptic effects, α2 receptors facilitate hypersynchronization and excitability. Dexmedetomidine suppresses α1 receptor-mediated tone (indirectly via locus coeruleus inhibition) while simultaneously enhancing α2 receptor signaling, particularly in the thalamus. This imbalance produces a distinct neural state described as “α2 wakefulness,” in which cortical EEG appears wake-like, but thalamocortical circuits exhibit heightened excitability and are prone to spike-wave discharges [41]. This model aligns with our findings, especially given the increased susceptibility observed in aged animals. Further supporting this mechanism, postsynaptic α2 receptors in the thalamus can directly drive oscillatory activity. Local thalamic microinjections of α2 agonists, such as clonidine and xylazine, have been shown to induce high-voltage spindles even in the absence of widespread norepinephrine suppression [42].

Finally, cortical α2 receptors, particularly in the prefrontal cortex, may modulate the amplitude and localization of these oscillations. α2 receptors in cortical pyramidal neurons and interneurons influence membrane potential and synaptic transmission, shifting network excitability toward states that favor synchronization. The prominent localization of high-voltage spikes to prefrontal cortex in our recordings likely reflects the combined effects of synchronized thalamic input and local cortical α2-receptor modulation within this region. Together, these findings suggest that dexmedetomidine-induced high-voltage spikes are mediated by both presynaptic and postsynaptic α2 adrenergic mechanisms and may be amplified by age-related changes in thalamocortical network dynamics.

### The aging brain and dexmedetomidine

Aging decreases sleep quality and EEG power, both of which are associated with decreased cognitive function [6, 31, 32]. Aging is also known to increase the sensitivity of the elderly to anesthetics. While many of the mechanisms behind these effects are not clear, they may overlap with mechanisms underlying the enhanced spiking in our aging animals. For example, both T-type CaV3.1 channels and hyperpolarization-activated cyclic nucleotide-gated channels of the thalamus have altered expression with aging, are associated with epilepsy, and are also critical for establishing the oscillatory activity associated with sleep.

Our data revealed significant differences in delta power (Figs. 3 and 4) and high-voltage spike characteristics (Figs. 5 and 6) between young-adult and aged mice. Older adults have more daytime sleepiness and significantly reduced amounts of NREM-sleep-associated slow-wave activity, which is vital for the restorative functions of sleep [43]. Broadband EEG power reduction is often seen with age, especially in lower frequencies, including delta power. Age-related declines in sleep quality and EEG power, particularly in lower frequencies like delta power, are well-documented and linked to cognitive decline [44]. Aging also increases sensitivity to anesthetics and sedatives like dexmedetomidine, which may exacerbate these effects [45]. This reduced wake-promoting activity and altered noradrenergic signaling in aged animals may explain the age-dependent differences we observed in dexmedetomidine’s effects.

### Implications for dexmedetomidine in seizure prone populations

The high-voltage spike bursts we observed resemble epileptiform activity, consistent with prior reports linking dexmedetomidine to such phenomena [41]. While dexmedetomidine does not typically induce seizures in healthy populations, it can unmask seizure susceptibility in individuals with a predisposition to seizures. Our findings are consistent with this, as we observed increased high-voltage spike bursts in aged mice, who are more susceptible to seizures compared to young adults [46, 47]. Age-related neuronal changes, including altered ion channel expression, increased oxidative stress, and reduced neurovascular resilience, may amplify this sensitivity. Other contributing factors include cerebrovascular disease [48], neurodegenerative pathology [49], and prior traumatic brain injury [50].

Norepinephrine plays a complex role in seizure regulation and dexmedetomidine reduces norepinephrine release [51, 52]. Several studies have shown that decreasing norepinephrine can lower seizure thresholds. For example, bilateral locus coeruleus lesions in rats reduce the electroshock seizure threshold [53], and dexmedetomidine lowers seizure threshold in both rodent epilepsy models [41, 54] and in cats anesthetized with enflurane [55]. Clinical observations mirror these findings: dexmedetomidine and its analog medetomidine have been implicated in seizures in a dog [56] and a human neonate [57]. Moreover, dexmedetomidine does not reliably suppress epileptiform discharges in humans and may even enhance them in patients with or without epilepsy [58]. In a study of dexmedetomidine for awake craniotomies, 5 of 27 patients experienced intraoperative seizures [59]. However, it is important to note that these observations in mice may not fully mirror clinical phenomena in humans, given well-established species differences in sleep–wake architecture, neural network dynamics, and drug pharmacokinetics [60–62]. Our findings should therefore be interpreted as preclinical evidence that illuminates underlying mechanisms rather than as direct clinical predictors. Nevertheless, rodent models provide a unique opportunity to dissect the cellular and circuit mechanisms by which dexmedetomidine alters brain activity across the lifespan, mechanisms that are inaccessible in human studies. By identifying these age- and susceptibility-dependent neural dynamics, our work lays the foundation for targeted hypotheses that can be tested in clinical populations.

Despite these risks, dexmedetomidine is frequently used in epilepsy resection surgeries due to its minimal suppression of cortical activity, which can help preserve seizure localization [63, 64]. In contrast, other anesthetics tend to suppress epileptic discharges and obscure epileptogenic zones. Taken together, these findings underscore the need for caution when using dexmedetomidine in older adults or individuals with a known seizure history or neurological comorbidities. While its unique neurophysiological profile can be advantageous in specific surgical contexts, clinicians should remain mindful of its potential to induce or unmask epileptiform activity in vulnerable populations.

### Implications for dexmedetomidine’s clinical use as a sleep aid

Although dexmedetomidine-induced sedation does not perfectly replicate natural sleep, it may still offer benefits in clinical settings, particularly in the intensive care unit. Low dose dexmedetomidine (which may avoid the incidence of high voltage spiking) has been shown to increase delta power during sedation, resembling recovery sleep after sleep deprivation. Consistent with this, a recent human study demonstrated that dexmedetomidine produces more sleep-like brain activity (delta power and network entropy) than propofol, with EEG patterns more closely approximating those seen during physiological NREM stage 2 sleep [65]. Studies suggest that dexmedetomidine may promote faster recovery with fewer complications compared to propofol in the intensive care unit [66–69]. Furthermore, low dose dexmedetomidine’s ability to increase NREM-like sleep in postoperative patients may help mitigate adverse events like delirium, cardiovascular complications, and prolonged mechanical ventilation [1, 70]. Therefore, while dexmedetomidine’s sedative state is not identical to natural sleep, it may still provide valuable therapeutic benefits over other general anesthetics in appropriate patient populations, particularly the intensive care unit and perioperative settings.

## Limitations and future directions

This study employed IP bolus administration of dexmedetomidine, which does not fully replicate the pharmacokinetics of the continuous low-dose intravenous infusions typically used in clinical practice. Rapid systemic absorption and high peak plasma concentrations following bolus IP dosing may transiently exaggerate neurophysiological effects, such as acute increases in spike activity and abrupt shifts in EEG power, compared to the more gradual and sustained EEG changes observed with intravenous infusions [71, 72]. These differences likely influence both the temporal dynamics and the magnitude of dexmedetomidine’s effects on brain activity. Alternative routes and dosing regimens, including low-dose intravenous or oral administration, may better approximate physiological sleep states. For example, oral dexmedetomidine has been reported to induce EEG patterns more closely resembling natural NREM sleep in mice, although REM sleep remains reduced [22]. Future studies using such dosing strategies may provide additional insight into the mechanisms underlying dexmedetomidine-induced brain states. A number of studies have observed dexmedetomidine-induced spectral changes in the EEG, as well as changes to sleep architecture and sleep-related phenomena such as spindles. In humans, spindles caused by dexmedetomidine are longer than NREM sleep spindles [18]. Additionally, due to the difficulty of detecting spindle activity with surface electrodes in mice, this study did not fully assess the role of spindles. Future studies could capture more sleep architecture dynamics with longer recordings, different routes of administration, and administration at different times of day.

A limitation of this study is the smaller sample size in the aged group compared to the young adult group. This reflects practical constraints inherent to aged animal studies, including limited availability and higher attrition rates. However, several factors support the validity of our findings despite this limitation: (1) robust nonparametric statistical approaches were used to minimize biases related to sample size differences; (2) age-related differences were large relative to within-group variability; and (3) multiple physiological measures showed convergent age-related changes. Future work with larger aged cohorts will further strengthen the generalizability of these findings.

The precise nature of the high-voltage spike events remains unresolved. While the sharp morphology of these waveforms resembles epileptiform discharges, similar events have been observed in wild-type rodents during NREM sleep and quiet wakefulness, and their origin remains uncertain. Such events have been variably interpreted as either absence-like discharges or normal features of rodent sleep EEG, and they occur predominantly during the light phase with an inverse relationship to locomotor activity [73]. Although we applied quantitative criteria to detect spikes and characterized their amplitude, width, and frequency, prior studies have shown that these metrics alone are often insufficient to reliably distinguish physiological from pathological spike–wave events due to substantial overlap in their features [74, 75]. Even though aged animals with evidence of major health impairments were excluded from the study, comprehensive standardized cognitive testing was not performed. However, aged mice were visually screened for alertness, grooming, weight dynamics and consistent baseline activity during pre-experimental acclimatization and baseline EEG recordings. Future studies combining high-density electrophysiology, behavioral correlates, and possibly genetic or pharmacological manipulations will be needed to determine whether these dexmedetomidine-induced spikes reflect a physiological spindle-like phenomenon or pathological epileptiform activity.

## Conclusion

Dexmedetomidine induced dose-dependent sedation, characterized by increased delta power, REM sleep suppression, and prominent high-voltage spike bursts, especially in aged mice. These spike bursts resemble high-voltage spindles and may reflect altered adrenergic tone, with aging increasing vulnerability to these oscillatory dynamics. While dexmedetomidine does not fully mimic natural sleep, it may still provide therapeutic benefits in specific patient populations, such as those in intensive care or postoperatively, when administered at low doses. However, its use in older or seizure-prone populations warrants careful consideration due to the increased risk of epileptiform activity.

## Data Availability

The datasets generated from the current study will be made available upon reasonable request.

## Abbreviations

ANOVA: Analysis of variance
ARRIVE: Animal Research: Reporting of In Vivo Experiments
CI: Confidence interval
DPSS: Discrete prolate spheroidal sequences
EEG: Electroencephalogram
EMG: Electromyogram
IP: Intraperitoneal
NREM: Non-rapid eye movement
REM: Rapid eye movement
